# The exploration of immune system function changes in marathon athletes after high-intensity training by Agent-Based Model

**DOI:** 10.3389/fspor.2026.1780114

**Published:** 2026-04-15

**Authors:** Hao Tian, Renzheng Zuo, Deng Wang, Guoping Qian, Qiang Ye, Adam Kawczyński, Robert Trybulski, Filipe Manuel Clemente

**Affiliations:** 1 Department of Physical Culture, Gdansk University of Physical Education and Sport, Gdańsk, Poland; 2 Nanjing Sport Institute, Nanjing, China; 3 Macao Polytechnic University, Macao, Macao SAR, China; 4 Department of Physical Education, Guangzhou Sport University, Guangzhou, China; 5 Faculty of Medicine, Wrocław University of Science and Technology, Wrocław, Poland; 6 Medical Department, Wojciech Korfanty Upper Silesian Academy in Katowice, Katowice, Poland; 7 Provita Żory Medical Center, Żory, Poland; 8 Department of Biomechanics, Gdansk University of Physical Education and Sport, Gdańsk, Poland; 9 Applied Research Institute (i2A), Polytechnic University of Coimbra, Coimbra, Portugal; 10 Sport Physical Activity and Health Research & Innovation Center, Coimbra, Portugal

**Keywords:** Agent-Based Model (ABM), high-intensity training, immune system function, marathon athletes, NetLogo

## Abstract

**Background:**

Marathon running imposes substantial metabolic demand. While the acute effects of exercise are well-documented, the chronic immunometabolic alterations associated with intensified training blocks in elite populations remain characterized by complex, non-linear dynamics. Contemporary debates persist regarding whether post-exercise lymphopenia represents immunosuppression (the “open window”) or a functional redistribution of immune effectors. This study explored the chronic effects of a four-week high-intensity training (HIT) block on immune markers in professional marathon athletes and evaluated the utility of an Agent-Based Model (ABM) for visualizing these system behaviors.

**Methods:**

Twenty-two professional marathon athletes (14 male, 8 female) underwent a four-week intensified training protocol characterized by sustained time in the severe-intensity domain (blood lactate > 7.0 mmol/L). Peripheral blood samples were analyzed pre- and post-training for leukocytes, immunoglobulins (Ig), cytokines (IL-6, IL-8, IL-10, TNF-α), and lymphocyte subsets. Concurrently, a NetLogo-based ABM was developed to simulate theoretical immune system dynamics under metabolic constraints.

**Results:**

The training period coincided with significant shifts in circulating immune markers. Total leukocyte counts and serum IgG levels were significantly lower post-training (*P* < 0.01). A marked inversion in T-cell homeostasis was observed, with the CD4+/CD8+ ratio decreasing to 0.98 (*P* < 0.01), driven by a reduction in CD4+ cells and maintenance of CD8+ populations. Cytokine analysis revealed a “resolution failure” profile: while pro-inflammatory markers IL-6, IL-8, and TNF-α increased (*P* < 0.01), the anti-inflammatory cytokine IL-10 was significantly reduced (*P* < 0.001). The ABM simulation qualitatively reproduced these emergent patterns, visualizing the non-linear contraction of T-cell populations consistent with the empirical data.

**Conclusions:**

A four-week block of high-intensity marathon training is associated with a state of immunometabolic perturbation characterized by reduced circulating leukocytes, CD4+/CD8+ imbalance, and an uncoupled inflammatory-resolution cytokine response. While plasma volume expansion may contribute to the observed lower cell concentrations, the specific suppression of IL-10 and CD4+ cells suggests a maladaptive response to chronic load. The agent-based model serves as an exploratory tool for visualizing potential immunological tipping points during intensified training, bridging the gap between reductionist data and complex system dynamics.

## 1 Introduction

As one of the most iconic long-distance endurance sports worldwide, marathon running requires athletes to possess exceptional aerobic metabolic capacity and resistance to fatigue (1). From a bioenergetic perspective, marathon competition is primarily performed within the heavy-intensity domain, corresponding to the physiological zone between the first ventilatory threshold (VT1) and the second ventilatory threshold (VT2) (2). Within this range, although blood lactate concentration remains elevated above resting levels, the body is able to sustain a dynamic balance between lactate production and clearance. However, in order to enhance competitive performance, professional athletes' training programs often incorporate substantial volumes of severe-intensity interval training. Such training exceeds VT2, resulting in significant disturbances to metabolic homeostasis and imposing cumulative physiological stress (3).

The metabolic cost of elite marathon running (approximately 600–800 kcal/h) imposes severe demands on substrate utilization (4). Under such high physiological load, skeletal muscle functions as a potent endocrine organ, secreting myokines (e.g., IL-6) that facilitate extensive crosstalk between metabolic and immune pathways (5). Historically, the prevailing dogma in exercise immunology—the “Open Window” theory—suggested that prolonged, severe-intensity training induces a transient, clinically significant decline in immune competency. This model posits that post-exercise lymphopenia and suppressed immunoglobulin secretion create a temporal window of heightened susceptibility to opportunistic infections, particularly upper respiratory tract infections (URTI) (6).

However, this “immunosuppression” paradigm has been vigorously challenged and refined in recent years. Emerging evidence, notably summarized by Campbell and Turner, argues that the lymphopenia observed following acute exercise may not reflect immune failure or cell death, but rather a functional redistribution of highly cytotoxic effector cells (e.g., NK cells, CD8+ T cells) from the blood to peripheral tissues—such as the lungs, gut, and Peyer's patches—to heighten barrier defense (7, 8). This reinterpretation frames the immune response to acute exercise as adaptive and regulatory, enhancing immune surveillance rather than diminishing it. Yet, a critical distinction must be made between acute adaptive redistribution and the consequences of sustained intensified training loads (Chronic Training Load) (9). When the recovery period is insufficient, the cumulative immunometabolic strain may shift the system from adaptive redistribution to maladaptive dysregulation, a transition that remains incompletely understood in elite cohorts (10).

Modern immunometabolism theory offers a novel mechanistic lens to resolve these conflicting perspectives. During high-intensity training (HIT), skeletal muscle and activated immune cells engage in intense competition for shared bioenergetic substrates, particularly glucose and glutamine (11). Activated T cells, akin to rapidly proliferating tumor cells, undergo metabolic reprogramming to rely on aerobic glycolysis (the Warburg effect) for clonal expansion (12). However, the systemic accumulation of lactate—often exceeding 7.0 mmol/L during elite marathon training—creates a complex regulatory environment. While high lactate concentrations can suppress glycolytic flux in T cells via negative feedback (13), recent breakthroughs in epigenetics have identified histone lactylation as a crucial regulatory mechanism (14).Under physiological conditions, intracellular lactate stimulates histone lactylation (e.g., H3K18la), which typically drives macrophages toward an anti-inflammatory (M2) phenotype and upregulates reparative cytokines like IL-10 (15). A failure in this lactylation-driven resolution mechanism—manifesting as high lactate load without the compensatory rise in IL-10—could signal a specific “resolution failure” phenotype characteristic of non-functional overreaching (NFOR).

Despite the biological complexity of these interactions, traditional sports science research often relies on reductionist approaches and linear statistical models that examine immune markers in isolation (16). The immune system is fundamentally a Complex Adaptive System (CAS), characterized by non-linearity, stochasticity, and emergent behavior. Traditional differential equation models struggle to capture the heterogeneous, spatial interactions of individual cells in a dynamic environment. In contrast, Agent-Based Modeling (ABM) simulates the autonomous behaviors and interaction rules of individual agents (cells) in a spatial environment to reproduce system-level patterns (17). While platforms like NetLogo are widely used in theoretical biology to simulate viral dynamics and tumor immunology, their application to quantifying the immunometabolic risks in human athletes remains a frontier of exploration (18).

Moreover, existing mechanistic studies often rely on animal models of “forced exercise,” which induce neuropsychological stress fundamentally different from the “voluntary training” of human athletes, limiting translational applicability (19). To address these theoretical and methodological gaps, the present study adopted a dual-pronged approach combining prospective empirical monitoring with computational simulation. The specific objectives of this study were: (1) to characterize the chronic immunometabolic phenotype of elite marathon runners following a four-week severe-intensity training block; (2) to quantify the magnitude of immune alterations using robust effect size estimation $\left(Cohen^{\prime }s\;d_{av}\right)$ and False Discovery Rate (FDR) correction; and (3) to evaluate the utility of an Agent-Based Model (ABM) in visualizing the non-linear emergent behaviors of the immune system under metabolic stress. We hypothesized that the HIT block would induce a specific immunometabolic signature characterized by “resolution failure” and that the ABM could serve as a qualitative heuristic tool to visualize the tipping points of this immune regulation.

## 2 Materials and methods

### 2.1 Study design and setting

This investigation was designed as a prospective, longitudinal, single-group pre–post intervention study. Twenty-two professional marathon athletes from the Shandong Provincial Team were followed across a four-week block of intensified high-intensity training (HIT). Immune and biochemical markers were assessed at two time points: one day before the start of the HIT block (baseline, T0) and 24 h after the final training session (post-intervention, T1), in order to characterize chronic (rather than acute) training-induced changes in immune function. The reporting of study design and conduct follows current CONSORT recommendations for transparent description of participant flow, timing of assessments, and study procedures for non-randomized intervention studies.

The study was conducted during the competitive preparation phase for the National Games (August 2023–August 2024 at the centralized training base of the Shandong Provincial Marathon Team in China, where athletes lived and trained under standardized conditions. All training sessions were supervised by professional coaches according to the predefined HIT protocol, and all blood collections were performed in the team's medical room in a controlled, morning fasted state (07:00–08:00).

### 2.2 Participants

We used a convenience sample of all eligible professional marathon athletes from the Shandong Provincial Team, who were approached consecutively during the 2023–2024 National Games preparation period and invited to participate in the study after screening against the inclusion and exclusion criteria. Twenty-two professional marathon athletes from the Shandong Provincial Team were recruited for this study. The cohort consisted of 14 males and 8 females with a mean age of 16.18 ± 2.83 years and a professional training history of 4–6 years. The study was conducted during the competitive preparation phase for the National Games (August 2023 to August 2024), a period characterized by high physiological demand.

Inclusion criteria were: (i) professional marathon athletes registered with the Shandong Provincial Team; (ii) in good physical condition, with no history of cardiovascular, hepatic, renal, or endocrine disease according to routine team medical examination; and (iii) able and willing to complete the planned 4-week high-intensity training block and all blood sampling procedures. To minimise confounding of immune outcomes, exclusion criteria were: (i) sports injuries requiring interruption of regular training in the preceding six months; (ii) current use of medications with potential immunomodulatory effects, including antibiotics, non-steroidal anti-inflammatory drugs (NSAIDs), or Traditional Chinese Medicine preparations; and (iii) symptoms or clinical diagnosis of upper respiratory tract infection or other acute infectious diseases within the two weeks preceding the baseline assessment.

Before entering this study, subjects were asked to provide informed consent after the aims of this study and the experimental protocol were explained by the researchers. The study was conducted according to the guidelines of the Declaration of Helsinki(1975), and approved by the institutional ethics committees of Nanjing Sport Institute, (Nanjing, China, 20220202003).

### 2.3 High-intensity training protocol

The intervention consisted of a four-week intensified “shock” training block designed to overload physiological capacities and stimulate specific adaptations in anaerobic threshold and lactate tolerance. The training regimen followed a polarized distribution but with a deliberately increased proportion of time spent in the severe-intensity domain (>Second Ventilatory Threshold, VT2).

To ensure the validity of the internal load quantification, Heart Rate (HR) was monitored continuously during all training sessions using the Polar Team Pro System (Polar Electro Oy, Kempele, Finland). This system utilizes a chest strap sensor (Polar H10) with a sampling frequency of 1000 Hz to detect R-R intervals. The device serves as a gold standard in field-based sports research, having shown a correlation of *r* = 0.996 when validated against clinical electrocardiograms (ECG). Data were extracted using the proprietary Polar Team Pro software for analysis of session average HR (HRmean), maximum HR (HRmax), and time spent in specific intensity zones.

Training Regimen and Physiological ResponsesThe 4-week protocol was structured into a progressive overload pattern. Key high-intensity sessions included:

(1) Interval Training (IT): Short to medium intervals (400 m–2,000 m) performed at intensities >95% VO2max to stimulate cardiac output and neuromuscular efficiency.

(2) Special Endurance (SE): Lactate tolerance training designed to push blood lactate (BLa) concentrations between 7.0–12.0 mmol/L, forcing the body to buffer high H+ concentrations.

(3) Tempo Runs (TR): Sustained efforts at the anaerobic threshold (170–180 bpm).

Blood lactate (BLa) was measured via fingertip capillary samples using a portable lactate analyzer (Lactate Scout 4, EKF Diagnostics, Germany) immediately after the main sets of high-intensity sessions (Tuesdays and Thursdays). The detailed weekly schedule and the physiological responses (Mean ± SD) recorded during the training block are presented in Table 1.

**Table 1:** Description of the 4-week high-intensity training (HIT) program and physiological responses.

Week	Day	Session Type	Training Content (Volume/Intensity)	Target Zone	Actual HR (bpm) (Mean ± SD)	BLa (mmol/L) (Mean ± SD)
W1	Mon	Recovery	12 km Easy Run (60–70% HRmax)	Moderate	142 ± 8	1.8 ± 0.4
	Tue	Intervals	15 × 400 m (Rec 60 s)	Severe	178 ± 6	8.2 ± 1.1
	Wed	Aerobic Base	20 km Steady State	Heavy	162 ± 5	3.1 ± 0.6
	Thu	Special End.	10 × 1,000 m (Rec 90 s)	Severe	181 ± 4	9.5 ± 1.3
	Fri	Recovery	10 km Easy Jog + Core Strength	Moderate	135 ± 9	1.5 ± 0.3
	Sat	Long Run	30 km (Mixed Pace)	Heavy	158 ± 7	2.8 ± 0.8
	Sun	Rest	Active Recovery/Massage	-	-	-
W2	Mon	Recovery	12 km Easy Run	Moderate	140 ± 6	1.6 ± 0.3
	Tue	Intervals	8 × 2,000 m (Rec 3 min)	Severe	176 ± 5	7.8 ± 0.9
	Wed	Aerobic Base	22 km Steady State	Heavy	164 ± 4	3.4 ± 0.5
	Thu	Lactate Tol.	4×(5 × 400 m) All-out	Extreme	188 ± 7	11.2 ± 1.5
	Fri	Recovery	10 km Easy Jog	Moderate	138 ± 8	1.4 ± 0.2
	Sat	Tempo Run	16 km at Threshold Pace	Heavy/Severe	172 ± 5	5.5 ± 1.0
	Sun	Rest	Total Rest	-	-	-
W3	Mon	Recovery	10 km Easy Run	Moderate	141 ± 7	1.7 ± 0.4
	Tue	Intervals	20 × 400 m (Rec 45 s)	Severe	182 ± 5	9.1 ± 1.2
	Wed	Aerobic Base	25 km Long Slow Distance	Heavy	155 ± 6	2.5 ± 0.5
	Thu	Special End.	5 × 3,000 m (Rec 4 min)	Severe	179 ± 6	8.5 ± 1.1
	Fri	Recovery	10 km Jog + Flexibility	Moderate	136 ± 5	1.6 ± 0.3
	Sat	Fartlek	20 km (1 min fast/1 min slow)	Mixed	168 ± 9	4.2 ± 1.2
	Sun	Rest	Total Rest	-	-	-
W4	Mon	Recovery	8 km Easy Run	Moderate	135 ± 6	1.3 ± 0.2
	Tue	Taper/Int.	6 × 1,000 m (Rec 2 min)	Severe	175 ± 4	6.8 ± 0.8
	Wed	Aerobic Base	12 km Steady State	Heavy	150 ± 5	2.2 ± 0.4
	Thu	Activation	3 × 2,000 m (Marathon Pace)	Heavy	168 ± 3	3.8 ± 0.6
	Fri	Rest	Total Rest (Pre-Test)	-	-	-
	Sat	Rest	Total Rest (Pre-Test)	-	-	-
	Sun	Post-Test	Sampling Day (T1)	-	-	-

HR, heart rate; BLa, blood lactate concentration; Rec, recovery duration. Values are presented as Mean ± Standard Deviation. The protocol was designed to maintain BLa > 7.0 mmol/L during severe intensity sessions.

### 2.4 Blood collection and biochemical analysis

Venous blood samples (5 mL) were collected from the antecubital vein in a fasting state (07:00–08:00) at two time points: one day before the start of the training block (Pre-training, T0) and 24 h after the final training session (Post-training, T1). The 24 h interval was selected to assess the chronic immune status rather than acute post-exercise leukocytosis.

**Figure 1:**
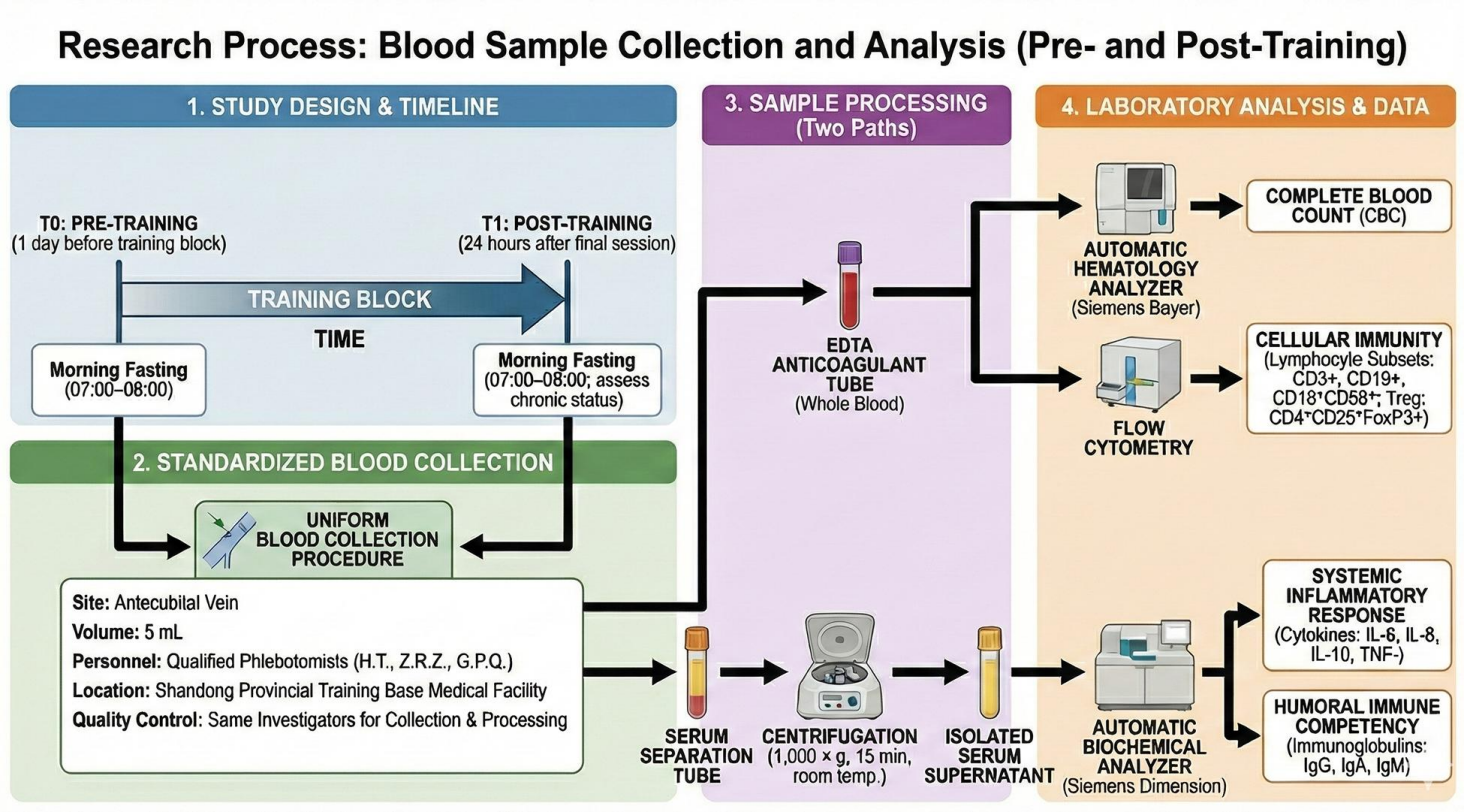
Schematic representation of the experimental design and study workflow.

Data Collection and Laboratory Quality Control: To ensure consistency and strict adherence to aseptic protocols, all blood collection procedures were performed by qualified phlebotomists, specifically the lead authors (H.T., Z.R.Z. and G.P.Q.), at the medical facility of the Shandong Provincial Training Base. The subsequent sample processing and laboratory analysis were also conducted by the same investigators (H.T., Z.R.Z. and G.P.Q.) to minimize inter-operator variability and ensure rigorous data treatment. The experimental workflow, illustrating the timeline of the training intervention, blood sampling points, and subsequent biochemical analyses, is presented in Figure 1.

Hematology and Lymphocyte Subsets: Blood samples collected in EDTA anticoagulant tubes were analyzed for complete blood count (CBC) utilizing an automatic six-part hematology analyzer (Siemens Bayer, USA). Peripheral blood lymphocyte subsets (including CD3+ T cells, CD19+ B cells, CD16 + CD56+ NK cells) and CD4 + CD25 + FoxP3+ Treg cells were quantified via flow cytometry to evaluate cellular immunity.

Serum Cytokines and Immunoglobulins: Additional samples were collected in serum separation tubes and centrifuged at 1,000 × g for 15 min at room temperature. The supernatant serum was isolated to quantify cytokines (IL-6, IL-8, IL-10, TNF-) and immunoglobulins (IgG, IgA, IgM) using an automatic biochemical analyzer (Siemens Dimension, USA). These markers were selected to assess the systemic inflammatory response and humoral immune competency.

### 2.5 Agent-Based modeling of immune dynamics

#### 2.5.1 Overview

To explore the non-linear system behaviors and potential “tipping points” of immune function under metabolic stress, we developed an Agent-Based Model (ABM) using the NetLogo 6.3 platform. The model description follows the ODD (Overview, Design concepts, Details) protocol (20) to ensure reproducibility and scientific rigor.

The primary purpose of this model is not precise clinical prediction of individual athletes, but rather to serve as a qualitative heuristic tool. It aims to simulate the emergent, non-linear dynamics of T-cell homeostasis when subjected to the dual constraints of high metabolic load (simulating HIT) and infection pressure.

Entities and State Variables: The model comprises three primary classes of agents:

Immune Agents: Representing T-cell populations (CD4+, CD8+) and Antigen-Presenting Cells (APCs). State variables include energy-level, activation-status (naïve/effector/exhausted), and location.

Pathogens: Representing opportunistic viral vectors. State variables include virulence and replication-rate.

Environment: A 2D spatial grid representing vascular and lymphatic compartments. Global variables include systemic-lactate (inhibitory factor) and glucose-availability (resource factor).

At each time step (tick), agents execute a cycle of: Movement → Metabolic Interaction (resource consumption) → Immune Interaction (detection/killing) → State Update (proliferation/apoptosis).

#### 2.5.2 Design concepts

Emergence: Systemic immunosuppression (the “crash”) is not explicitly hard-coded. Instead, it emerges from the competition for limited energetic substrates between the high metabolic demand of the “training load” (simulated as resource depletion) and the energetic cost of T-cell proliferation.

Sensing and Adaptation: Agents sense local lactate concentrations. High lactate levels (mimicking the post-HIT environment) trigger a suppression-threshold in T-cells, inhibiting their proliferation logic based on the Warburg effect constraints.

Stochasticity: Interactions between immune agents and pathogens are probabilistic, reflecting the biological noise inherent in immune surveillance.

#### 2.5.3 Details and algorithms

Initialization: The model is initialized using mean parameters derived from the empirical Pre-Training (T0) dataset of the 22 athletes (e.g., initial ratios of CD4/CD8).Multi-Objective Optimization (MOP): To simulate the trade-off immune cells face between “survival” and “effector function” under stress, agent decision-making is governed by a Multi-Objective Optimization Problem (MOP) algorithm (29). The optimal behavior strategy *y* for an agent is solved as:

$$\min y=f(x)=[\;f_{1}(x),f_{2}(x),L,f_{\pi }(x)]$$ (1)

Subject to metabolic constraints:

$$g_{i}(x)\leq 0,i=1,2,\ldots ,q$$ (2)

Where *x* represents the decision vector (e.g., proliferate vs. rest) and $g_{i}(x)$ represents the physiological constraints (e.g., available glucose < threshold). The model seeks a Pareto optimal set $\left(B^{*}\right)$ where immune defense is maximized without exceeding the organism's metabolic capacity:

$$B^{*}=\{x^{*}:x\in X_{f}:xf\;x^{*}\}$$ (3)

Submodels: A Non-dominated Neighbor Immune Algorithm (NNIA) was implemented to simulate the clonal expansion of specific T-cell subsets (antibodies) in response to antigen recognition, modulated by the global stress variables.

#### 2.5.4 Model validation

Given the lack of a separate validation dataset, the model was validated using Pattern-Oriented Modeling (POM) criteria. The model was deemed valid if it could qualitatively reproduce three key structural patterns observed in the empirical post-training data: (1) the non-linear decay of total leukocyte counts; (2) the inversion of the CD4+/CD8+ ratio; and (3) the failure of inflammatory resolution (sustained pathogen load) under high-intensity parameters.

### 2.6 Sample size

To ensure the statistical power of this study, we performed sample size calculations using G*Power software. The parameters used for the calculation are as follows:

1. Effect size (d): 0.8, indicating a large expected effect (Cohen's d).
2. Significance level (αerr prob): 0.05, corresponding to a 5% probability of a Type I error.
3. Statistical power (Power, 1-βerr prob): 0.8, meaning we aim for an 80% chance of detecting an effect if it exists.

Based on these parameters, G*Power calculated a required sample size of 19 participants. This indicates that, with the expected effect size and statistical power, 19 participants would be sufficient for the study.

#### 2.6.1 Sample size calculation formula

The sample size for a paired *t*-test is commonly estimated using the following formula:

$$N\approx {\left(\frac{Z_{\alpha /2}+Z_{\beta }}{d_{z}}\right)}^{2}+\;3$$

$$N\approx {\left(\frac{1.96+0.84}{0.8}\right)}^{2}+\;3\approx 19$$

Where:

$Z_{\alpha /2}=1.96$ (the critical value for *α* = 0.05 in a two-tailed test)
$Z_{\beta }=0.84$ (the critical value for *β* = 0.20, corresponding to 80% power)
$d_{z}$ is the effect size (in this study, 0.8)

Substituting these values into the formula, the calculated sample size is 19.

This sample size is adequate to detect large effects and ensures that the study has 80% statistical power. Although the calculated sample size is 19, the final study sample was increased to 22 participants to ensure robustness and to account for potential participant variability.

### 2.7 Statistical analysis

Data analysis was performed using Jamovi (Version 2.3) and the R statistical computing environment. Descriptive statistics for all immunometabolic variables are presented as Mean ± Standard Deviation (SD). The normality of data distribution was assessed using the Shapiro–Wilk test. For variables satisfying the assumption of normality, paired samples *t*-tests were employed to evaluate intra-group differences (Pre- vs. Post-training); otherwise, the non-parametric Wilcoxon signed-rank test was utilized.

Correction for Multiple Comparisons: Given the exploratory nature of the broad immune panel assessed in this study (k=17) dependent variables), uncorrected univariate testing carries a high risk of Type I errors. To address this, we applied the Benjamini-Hochberg procedure to control the False Discovery Rate (FDR). Adjusted *p*-values (*q*-values) were calculated for all secondary exploratory outcomes (cytokines, leukocyte subsets, and immunoglobulins), with statistical significance accepted at *q* < 0.05. The CD4+/CD8+ ratio was designated *a priori* as the primary outcome measure of immune homeostasis (21).

Effect Size Calculation: To accurately quantify the magnitude of physiological changes while avoiding the overestimation often inherent in repeated-measures designs with high intra-subject correlations (*r* > 0.8), we reported Cohen's $d_{\mathrm{av}}$ (Standardized Mean Difference based on average variance) rather than Cohen's $d_{\mathrm{av}}$ (22). Effect sizes were calculated using R based on the following formula (23):

$$d_{av}=\frac{M_{\mathrm{post}}-M_{\mathrm{pre}}}{\frac{SD_{\mathrm{pre}}+SD_{\mathrm{post}}}{2}}$$

where M represents the mean and SD represents the standard deviation at baseline (pre) and post-intervention (post). The magnitude of $d_{\mathrm{av}}$ was interpreted using thresholds specific to sports science research: trivial (<0.2), small (0.2–0.6), moderate (0.6–1.2), large (1.2–2.0), and very large (>2.0) (24).

## 3 Results

Physiological Response to Training Load All 22 athletes successfully completed the four-week intensified training block. During the key high-intensity sessions (Interval Training and Special Endurance), the mean heart rate was maintained between 175 and 190 bpm, and post-session blood lactate concentrations consistently ranged between 7.0 and 12.0 mmol/L (Table 1), confirming that the training load successfully targeted the severe-intensity domain (>VT2).

### 3.1 Changes in total leukocytes and subsets

**Figure 2:**
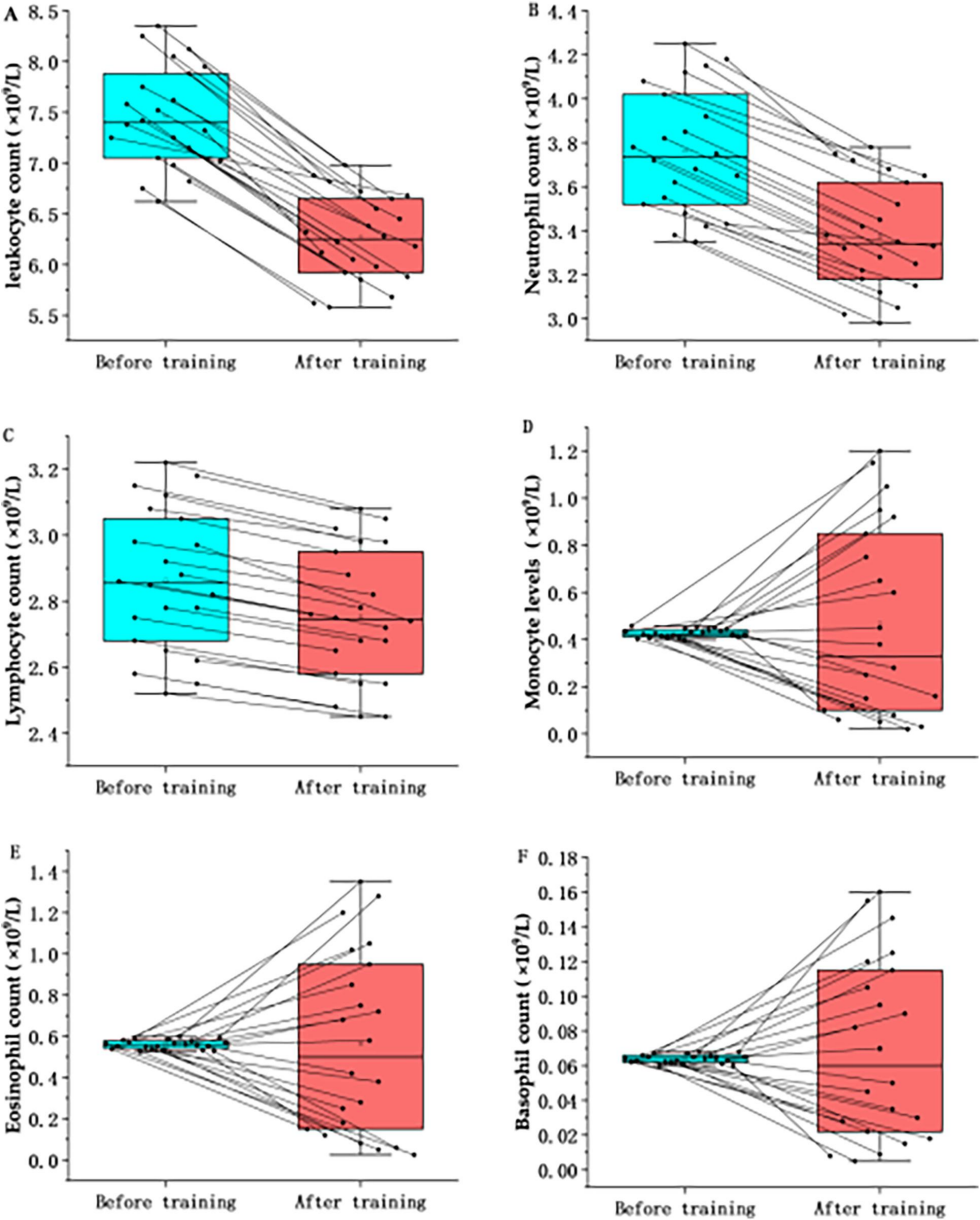
Effects of high-intensity training on leukocyte classification in marathon athletes. **(A)** The comparison of the leukocyte count in marathon athletes before and after high-intensity training; **(B)** The comparison of the Neutrophil count in marathon athletes before and after high-intensity training; **(C)** The comparison of the Lymphocyte count in marathon athletes before and after high-intensity training; **(D)** The comparison of the Monocyte count in marathon athletes before and after high-intensity training; **(E)** The comparison of the Eosinophil count in marathon athletes before and after high-intensity training; **(F)** The comparison of the Basophil count in marathon athletes before and after high-intensity training.

The training block coincided with a widespread reduction in circulating leukocyte counts (Table 2; Figure 2). Total leukocyte counts decreased significantly from pre-training $\left(7.32\pm 0.60\times 10^{9}/L\right)$ to post-training $\left(6.13\pm 0.50\times 10^{9}/L,\right)$ P<0.001), with a very large effect size $\left(Cohen^{\prime }s\;d_{av}=2.64\right)$. This decline was driven primarily by significant reductions in the absolute counts of neutrophils $\left(q<0.01,d_{av}=1.49\right)$ and lymphocytes $\left(q<0.01,d_{av}=0.80\right)$. No statistically significant changes were observed in monocyte, eosinophil, or basophil counts after FDR correction (q>0.05).

**Table 2:** Statistical differences in total leukocyte count and classification before and after training.

Variables	N	Before training	After training	Changes	*p*	*q*-value	$\mathrm{Cohe}n^{\prime }s\;d_{\mathrm{av}}$
Leukocyte	22	7.32 ± 0.60	6.13 ± 0.50	−1.19 ± 0.78	<0.001	.0012*	2.64
Neutrophil	22	3.70 ± 0.31	3.33 ± 0.28	−0.37 ± 0.42	<0.001	.0012*	1.49
Lymphocyte	22	2.81 ± 0.32	2.71 ± 0.22	−0.10 ± 0.39	<0.001	.0012*	0.8
Monocyte	22	0.42 ± 0.02	0.37 ± 0.37	−0.05 ± 0.37	>0.05	.743	−0.2
Eosinophil	22	0.56 ± 0.03	0.47 ± 0.47	−0.09 ± 0.47	>0.05	.975	0.04
Basophil	22	0.06 ± 0.003	0.05 ± 0.05	−0.01 ± 0.05	>0.05	.743	−0.54

Data represented as mean ± SD; *P*: Unadjusted significance level from Wilcoxon signed-rank test; *q*-value: Adjusted *P* calculated using the Benjamini-Hochberg False Discovery Rate (FDR) procedure to correct for multiple comparisons. Statistical significance is defined as *q* < 0.05; Cohen's $d_{\mathrm{av}}$: Standardized effect size calculated using the average standard deviation of both time points $\left(d_{\mathrm{av}}=M_{\mathrm{diff}}/SD_{\mathrm{avg}}\right)$, rather than difference scores $\left(d_{z}\right)$, to correct for overestimation in repeated measures; Interpretation: Effect size magnitudes are interpreted as: trivial (<0.2), small (0.2–0.6), moderate (0.6–1.2), large (1.2–2.0), and very large (>2.0).

*Statistical significance after FDR correction (*q* < 0.05).

### 3.2 Alterations in Serum immunoglobulins

**Figure 3:**
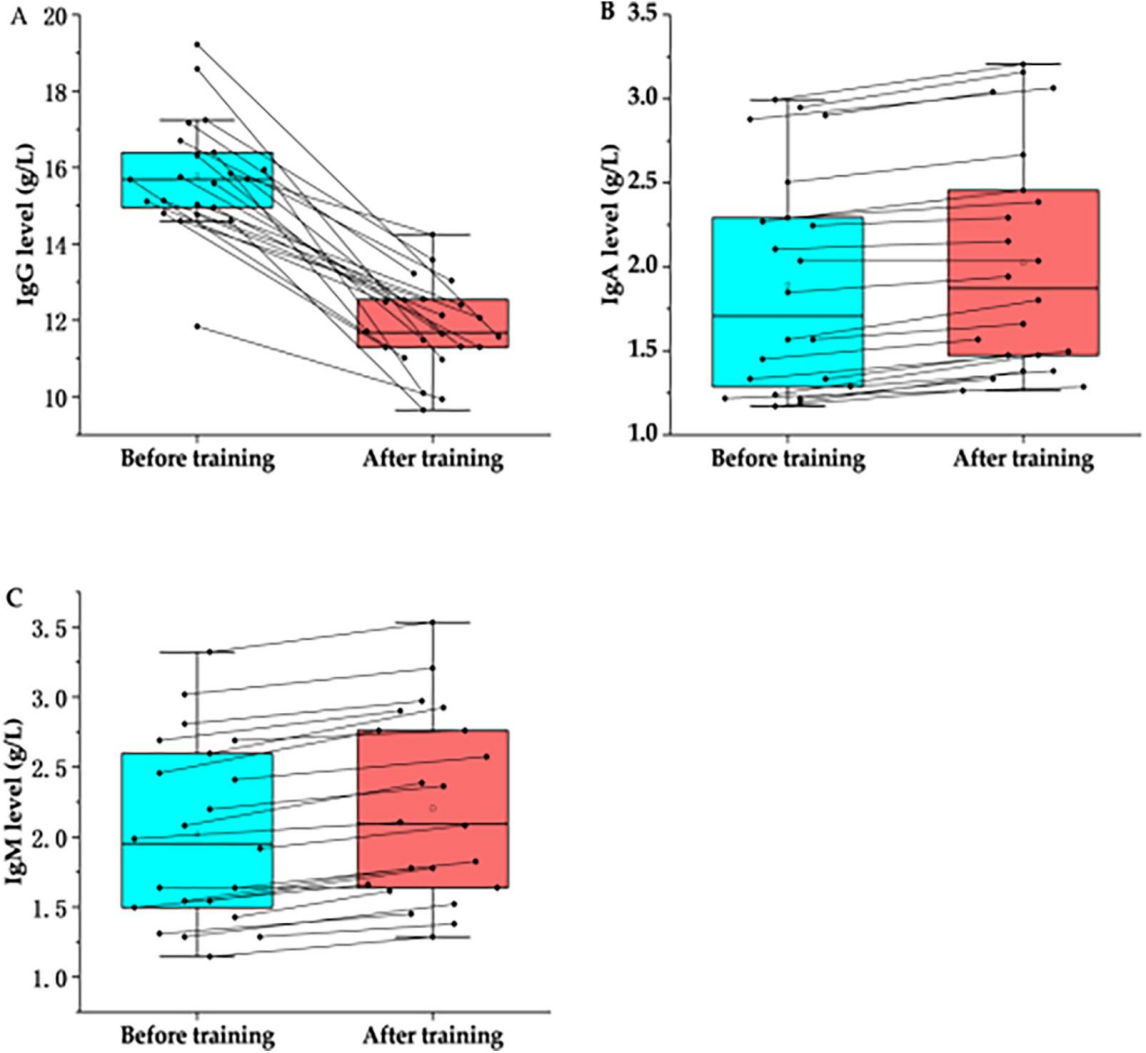
Effects of high-intensity training on Ig in marathon athletes. **(A)** The comparison of Ig G levels in marathon athletes before and after training; **(B)** The comparison of Ig A levels in marathon athletes before and after training; **(C)** The comparison of Ig M levels in marathon athletes before and after training.

Serum immunoglobulin analysis revealed distinct lineage-specific responses (Table 3; Figure 3). Serum IgG levels decreased significantly post-training (P<0.001,q<0.01), although the effect size was small to moderate $\left(d_{av}=0.31\right)$ compared to cellular changes. Conversely, IgM levels showed a significant increase $\left(P<0.001,d_{av}=2.95\right)$, while IgA levels exhibited a minor increase $\left(P<0.001,d_{av}=0.35\right)$. These contrasting trends suggest a differential regulation of humoral immunity classes under chronic training stress.

**Table 3:** Statistical differences in Ig before and after training.

Variables	*N*	Before training	After training	Changes	*p*	*q*-value	Cohen's $d_{\mathrm{av}}$
Lg A	22	1.8905 ± 0.6521	2.0234 ± 0.6664	0.1329 ± 0.0647	<0.001	.0012*	−0.35
Lg G	22	15.7706 ± 1.5071	11.8295 ± 1.1554	−3.9411 ± 1.6022	<0.001	.0012*	−0.31
Lg M	22	2.0223 ± 0.6415	2.2041 ± 0.6627	0.1818 ± 0.0767	<0.001	.0012*	2.95

Data represented as mean ± SD; *P*: Unadjusted significance level from Wilcoxon signed-rank test; *q*-value: Adjusted *P* calculated using the Benjamini-Hochberg False Discovery Rate (FDR) procedure to correct for multiple comparisons. Statistical significance is defined as *q* < 0.05; Cohen's $d_{\mathrm{av}}$: Standardized effect size calculated using the average standard deviation of both time points $\left(d_{\mathrm{av}}=M_{\mathrm{diff}}/SD_{\mathrm{avg}}\right)$, rather than difference scores $\left(d_{z}\right)$, to correct for overestimation in repeated measures; Interpretation: Effect size magnitudes are interpreted as: trivial (<0.2), small (0.2–0.6), moderate (0.6–1.2), large (1.2–2.0), and very large (>2.0).

*Statistical significance after FDR correction, with *q* < 0.05.

### 3.3 Cytokine profile: pro-inflammatory shift and IL-10 suppression

**Figure 4:**
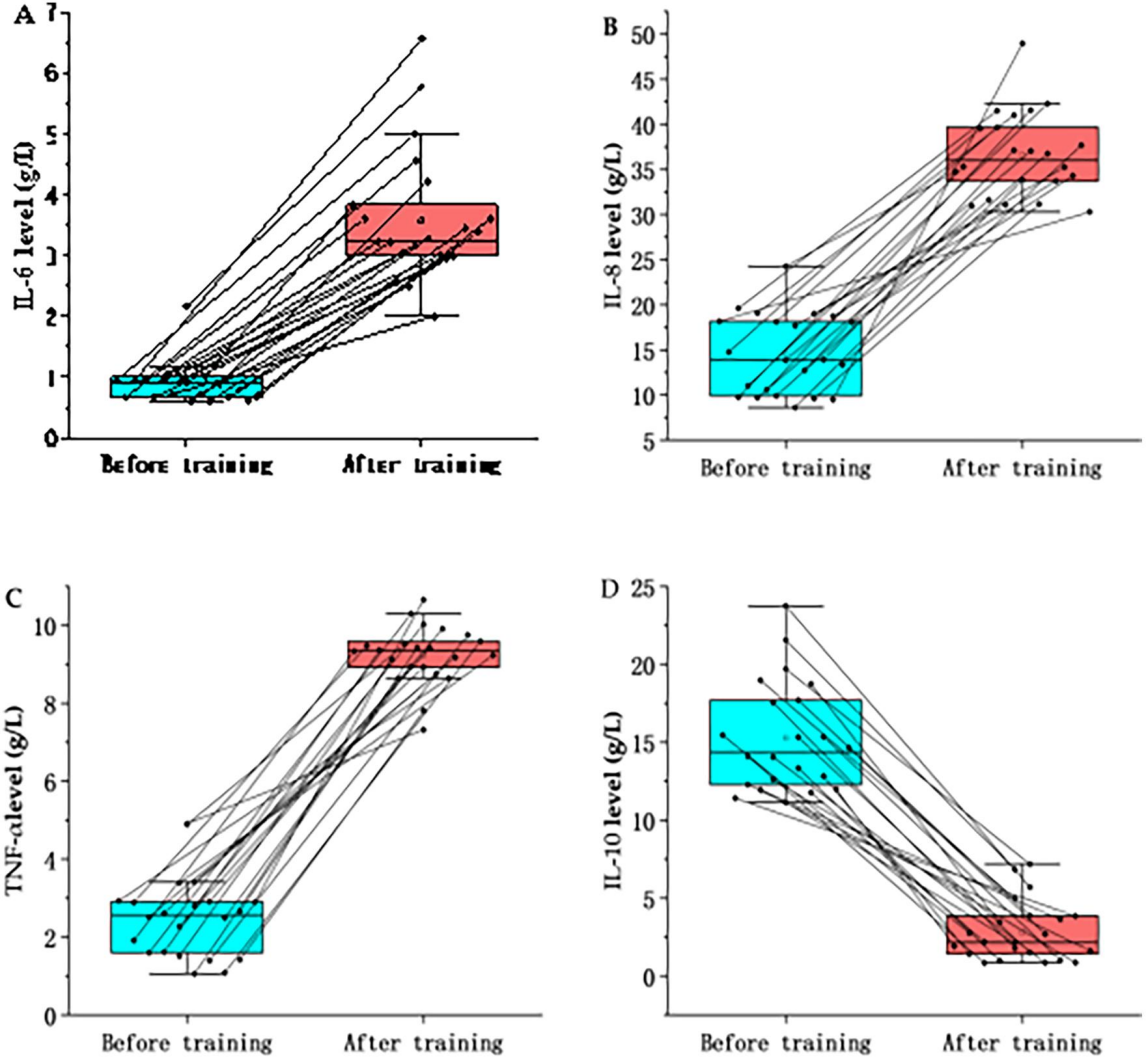
Effects of high-intensity training on cytokines in marathon athletes. The comparison of IL-6 levels in marathon athletes before and after training; **(B)** The comparison of IL-8 levels in marathon athletes before and after training; **(C)** The comparison of TNF- α levels in marathon athletes before and after training; **(D)** The comparison of IL-10 levels in marathon athletes before and after training.

The cytokine response was characterized by a marked uncoupling of pro- and anti-inflammatory markers (Table 4; Figure 4). We observed significant elevations in pro-inflammatory cytokines, including IL-6 $\left(d_{av}=3.50\right)$, IL-8 $\left(d_{av}=4.85\right)$, and TNF-α $\left(d_{av}=7.44\right)$ (all P<0.001,q<0.01). In contrast, the anti-inflammatory cytokine IL-10 decreased substantially from 15.28±3.54pg/mLto2.83±1.92pg/mL(P<0.001,q<0.01), yielding a large effect size $\left(d_{av}=4.77\right)$.

**Table 4:** Statistical differences in cytokines before and after training.

Variables	N	Before training	After training	Changes	*p*	*q*-value	Cohen's $d_{\mathrm{av}}$
IL-6	22	0.9015 ± 0.3324	3.5758 ± 1.0802	2.6742 ± 0.9545	<0.001	.0012*	-3.5
IL-8	22	14.5546 ± 4.4355	36.6076 ± 4.6686	22.0530 ± 6.8393	<0.001	.0012*	−4.85
TNF-α	22	2.5108 ± 1.0542	9.2420 ± 0.7456	6.7312 ± 1.4342	<0.001	.0012*	−7.44
IL-10	22	15.2823 ± 3.5390	2.8291 ± 1.9216	−12.4532 ± 2.5659	<0.001	.0012*	4.77

Data represented as mean ± SD; *P*: Unadjusted significance level from Wilcoxon signed-rank test; *q*-value: Adjusted *P* calculated using the Benjamini-Hochberg False Discovery Rate (FDR) procedure to correct for multiple comparisons. Statistical significance is defined as *q* < 0.05; Cohen's $d_{\mathrm{av}}$: Standardized effect size calculated using the average standard deviation of both time points $\left(d_{\mathrm{av}}=M_{\mathrm{diff}}/SD_{\mathrm{avg}}\right)$, rather than difference scores $\left(d_{z}\right)$, to correct for overestimation in repeated measures; Interpretation: Effect size magnitudes are interpreted as: trivial (<0.2), small (0.2–0.6), moderate (0.6–1.2), large (1.2–2.0), and very large (>2.0).

*Statistical significance after FDR correction, with *q* < 0.05.

### 3.4 Restructuring of lymphocyte subsets

**Figure 5:**
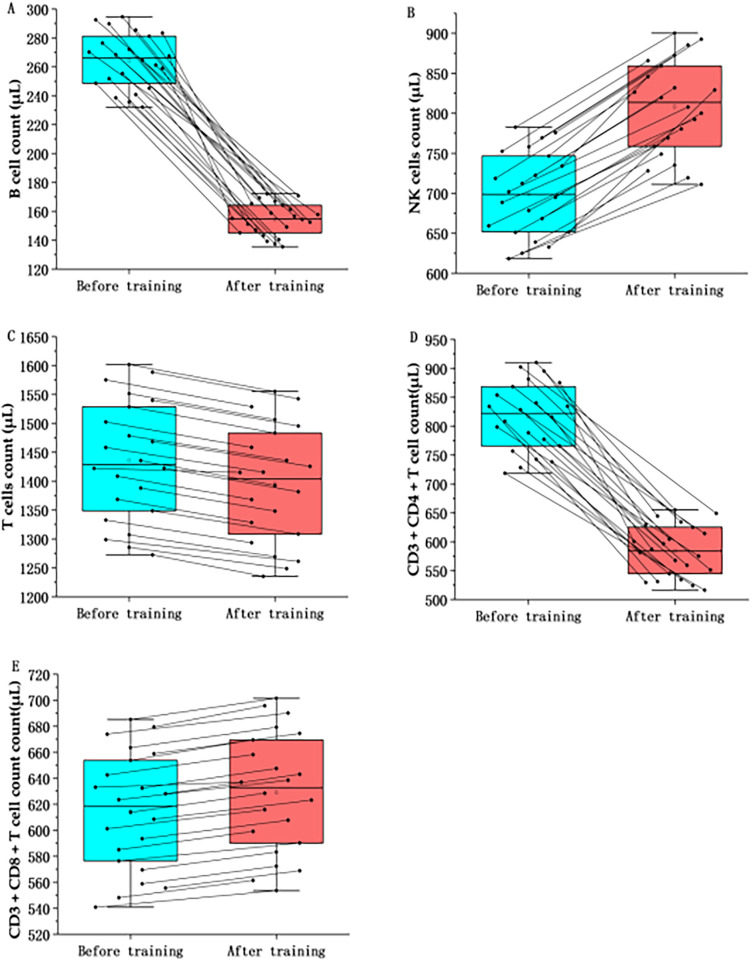
Effects of high-intensity training on peripheral blood lymphocyte subsets in marathon athletes. **(A)** The comparison of the B cell count in marathon athletes before and after high-intensity training; **(B)** The comparison of the NK cells count in marathon athletes before and after high-intensity training; **(C)** The comparison of the T cell count in marathon athletes before and after high-intensity training; **(D)** The comparison of the CD3 + CD4 + T cell count in marathon athletes before and after high-intensity training; **(E)** The comparison of the CD3 + CD8 + T cell count in marathon athletes before and after high-intensity training.

Flow cytometric analysis indicated a significant restructuring of the lymphoid compartment (Table 5; Figure 5). The primary outcome measure, the CD4+/CD8+ T-cell ratio, inverted significantly from a baseline of 1.35 ± 0.29–0.98 ± 0.18 post-training (P<0.01). This inversion was driven by a significant reduction in CD4+ Helper T cells $\left(d_{av}=4.41\right)$ and a concomitant increase in NK cells $\left(d_{av}=1.89\right)$, while CD8+ Cytotoxic T cells remained relatively stable $\left(d_{av}=0.32\right)$. B cells (CD19+) exhibited the most pronounced decline among all subsets, dropping by approximately 41% $\left(d_{av}=7.10\right)$.

**Table 5:** Statistical differences in peripheral blood lymphocyte subsets before and after training.

Variables	N	Before training	After training	Changes	*p*	*q*-value	Cohen's $d_{\mathrm{av}}$
B cell	22	263.19 ± 19.47	153.68 ± 11.37	−109.51 ± 22.54	<0.001	.0012*	7.1
NK cells	22	698.52 ± 51.67	803.63 ± 59.44	105.11 ± 78.85	<0.001	.0012*	−1.89
T cell	22	1,429.26 ± 105.71	1,387.96 ± 102.66	−41.30 ± 147.38	<0.001	.0012*	0.4
CD3 + CD4 + T cell	22	811.92 ± 60.05	584.35 ± 43.22	−227.57 ± 73.96	<0.001	.0012*	4.41
CD3 + CD8 + T cell	22	611.08 ± 45.20	625.96 ± 46.28	14.88 ± 64.64	<0.001	.0012*	−0.32

Data represented as mean ± SD; *P*: Unadjusted significance level from Wilcoxon signed-rank test; *q*-value: Adjusted *P* calculated using the Benjamini-Hochberg False Discovery Rate (FDR) procedure to correct for multiple comparisons. Statistical significance is defined as *q* < 0.05; Cohen's $d_{\mathrm{av}}$: Standardized effect size calculated using the average standard deviation of both time points $\left(d_{\mathrm{av}}=M_{\mathrm{diff}}/SD_{\mathrm{avg}}\right)$, rather than difference scores $\left(d_{z}\right)$, to correct for overestimation in repeated measures; Interpretation: Effect size magnitudes are interpreted as: trivial (<0.2), small (0.2–0.6), moderate (0.6–1.2), large (1.2–2.0), and very large (>2.0).

*Statistical significance after FDR correction, with *q* < 0.05.

### 3.5 Agent-based model simulation

**Figure 6:**
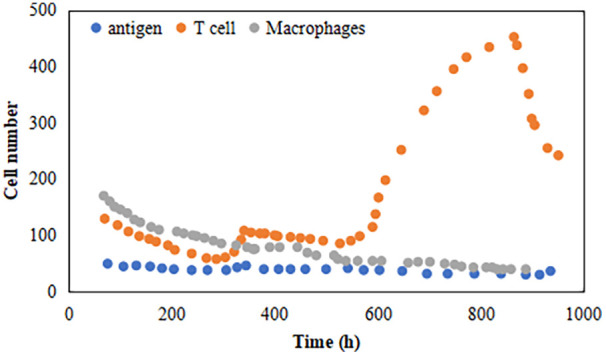
The simulation curve of the immune response in the model.

The ABM was initialized with parameters derived from the empirical pre-training data. The simulation outputs qualitatively reproduced the non-linear dynamics observed in the biological data (Figure 6). Specifically, under conditions simulating high metabolic substrate competition (high lactate/low glucose), the virtual T-cell population exhibited a rapid, non-linear contraction phase similar to the empirical reduction in CD4+ cells. The model visualized a system-level “tipping point” where immune resilience (represented by agent density) declined sharply once metabolic stress exceeded a critical threshold.

## 4 Discussion

The primary aim of this study was to characterize the chronic immunometabolic phenotype of elite marathon athletes following a four-week intensified training block. Our results reveal a complex physiological state characterized by substantial reductions in circulating leukocyte subsets, a marked inversion of the CD4+/CD8+ ratio, and a cytokine profile indicative of “resolution failure”—specifically, elevated pro-inflammatory mediators concurrent with a profound suppression of the anti-inflammatory cytokine IL-10. Importantly, by applying robust effect size estimation $\left(d_{av}\right)$ and FDR correction, we confirmed that these alterations represent statistically significant and large-magnitude shifts $\left(d_{av}>0.8\right)$ (21, 22), rather than trivial fluctuations. While the NetLogo agent-based model successfully visualized the non-linear “tipping points” of these dynamics, the biological interpretation of these data requires a nuanced perspective that integrates concepts of immune redistribution, hemodilution, and metabolic regulation.

### 4.1 Reinterpreting lymphopenia: immunosuppression or redistribution?

A pivotal finding was the widespread reduction in circulating lymphocyte counts, particularly B cells $\left(d_{\mathrm{av}}=7.10\right)$ and CD4+ T cells $\left(d_{\mathrm{av}}=4.41\right)$. Historically, such “lymphopenia” has been interpreted as a direct sign of immunosuppression or cell death (the “Open Window” theory) (25). However, our data align more closely with the contemporary “Redistribution Hypothesis” proposed by Campbell and Turner (7). Exercise-induced elevations in catecholamines and glucocorticoids are known to mobilize effector cells (NK cells and CD8+ T cells) into the circulation, which subsequently extravasate to peripheral tissues—such as the lungs, gut, and Peyer's patches—to conduct mucosal surveillance. In our cohort, the observation that CD8+ T cells were relatively preserved ($d_{\mathrm{av}}=0.32$, non-significant) while CD4+ cells and B cells were depleted suggests a differential mobilization pattern. The “disappearance” of cells from the blood likely reflects their functional redeployment to potential sites of pathogen entry rather than systemic failure. Thus, the observed CD4+/CD8+ ratio inversion (dropping to 0.98) may not indicate a collapse of immunity, but rather a strategic shift towards cytotoxic surveillance in peripheral tissues during periods of high physiological stress.

### 4.2 The confounder of plasma volume expansion (Pseudo-leukopenia)

Critically, we must address the influence of hemodynamics on the interpretation of concentration-based markers. It is well-established that chronic endurance training induces plasma volume expansion (PVE), which can range from 10% to 20% in elite athletes. This hypervolemia inevitably leads to a dilutional effect known as “pseudo-leukopenia” (26). As we did not correct for PVE using hemoglobin/hematocrit shifts (a limitation of this study), a portion of the observed reductions in total leukocytes and IgG $\left(d_{av}=0.31\right)$ is undoubtedly attributable to hemodilution.

However, dilution alone cannot explain the magnitude of specific cellular shifts. If PVE were the sole driver, we would expect a uniform ∼10%–15% decrease across all subsets. Instead, we observed a massive ∼40% reduction in B cells and a ∼28% reduction in CD4+ T cells, contrasting with stable or increased NK/CD8+ populations. This heterogeneity confirms that true biological modulation—likely driven by cortisol-mediated apoptosis or tissue migration—is occurring superimposed upon the background of hemodilution.

### 4.3 Metabolic regulation and the “resolution failure” phenotype

Perhaps the most striking and novel finding of this study is the cytokine profile. We observed a classic exercise-induced elevation in pro-inflammatory markers (IL-6, TNF−α, IL-8), which is typically balanced by a compensatory rise in anti-inflammatory IL-10. In our cohort, however, IL-10 was paradoxically suppressed $\left(P<0.001,d_{av}=4.77\right)$ (27). This uncoupling suggests a phenomenon of “Inflammatory Resolution Failure”. Recent mechanistic studies in 2024–2025 link this to immunometabolic dysregulation. Under normal physiological conditions, lactate derived from exercise promotes histone lactylation (e.g., H3K18la) in macrophages, driving an M2-like anti-inflammatory phenotype and IL-10 production (14, 15). The failure to upregulate IL-10 in our athletes, despite high training loads (and presumably high lactate flux), suggests a breakdown in this lactylation-driven epigenetic feedback loop. We hypothesize that chronic, severe-intensity training may lead to “substrate exhaustion” or epigenetic enzymatic fatigue (e.g., p300/CBP dysfunction), preventing the resolution of inflammation and leaving athletes in a state of persistent low-grade inflammation and fatigue—a hallmark of Non-Functional Overreaching (NFOR) (28).

### 4.4 Limitations

First, the single-group pre-post design precludes definitive causal inference; we cannot rule out seasonal or environmental influences on immune markers.

Second, it is well-established that high-intensity endurance training induces plasma volume expansion (PVE), typically ranging from 10% to 20% (Convertino, 1991; Girard et al., 2024). A major limitation of the present study is the lack of direct correction for PVE using hemoglobin and hematocrit shifts. Consequently, a portion of the observed reductions in leukocyte subsets and immunoglobulins likely reflects hemodilution (pseudo-leukopenia) rather than absolute cellular depletion. However, the magnitude of the decrease observed in B cells (−41%) and CD4+ T cells (−28%) exceeds the theoretical maximum contribution of PVE (∼20%), suggesting that while hemodilution contributes to the observed profile, it does not fully account for the pronounced specific immune alterations, particularly the inversion of the CD4+/CD8+ ratio.

Third, we measured immune status in peripheral blood only; without tissue biopsies or specific migration markers (e.g., CX3CR1), the redistribution hypothesis remains inferential. Finally, the sample size (*n* = 22), while typical for elite athlete cohorts, limits the generalization of findings to broader populations.

### 4.5 Conclusions

In summary, a four-week block of severe-intensity marathon training is associated with profound immunometabolic perturbations. Beyond the expected effects of hemodilution and redistribution, our data highlight a specific failure of inflammatory resolution mechanisms (IL-10 suppression) and a significant restructuring of the T-cell compartment. These findings challenge the simple “immunosuppression” narrative and point towards a complex model of metabolic-epigenetic dysregulation. Future research should integrate transcriptomics to investigate histone lactylation pathways in athletes and validate ABM tools for personalized load monitoring.

## Data Availability

The data supporting the findings of this study are available from the corresponding author upon reasonable request.
